# Exacerbated Hepatic Stress and Systemic Metabolic Disruption Driven by SARS‐CoV‐2 P.1 Variant in K18‐hACE2 Mice

**DOI:** 10.1002/iid3.70457

**Published:** 2026-06-03

**Authors:** Monara Kaélle Sérvulo Cruz Angelim, Patrícia Brito Rodrigues, Antônio Thiago Pereira Campos, Giovanni Freitas Gome, Gabriela Fabiano Souza, Valquíria Aparecida Matheus, Carlos Lenz Cesar, José Luiz Proença‐Módena, Marco Aurélio R. Vinolo, Pedro M. Moraes‐Vieira

**Affiliations:** ^1^ Laboratory of Immunometabolism, Department of Genetics, Microbiology and Immunology, Institute of Biology University of Campinas (UNICAMP) Campinas São Paulo Brazil; ^2^ Laboratory of Immunoinflammation, Department of Genetics, Microbiology and Immunology, Institute of Biology University of Campinas (UNICAMP) Campinas São Paulo Brazil; ^3^ Physics Department Federal University of Ceará (UFCE) Fortaleza Ceará Brazil; ^4^ Ribeirão Preto Medical School, Center of Research in Inflammatory Diseases University of São Paulo Ribeirão Preto São Paulo Brazil; ^5^ Laboratory of Emerging Viruses, Department of Genetics, Microbiology and Immunology, Institute of Biology University of Campinas (UNICAMP) Campinas São Paulo Brazil; ^6^ Advanced Therapies Laboratory, Faculty of Pharmaceutical Sciences University of Campinas (UNICAMP) Campinas São Paulo Brazil; ^7^ Experimental Medicine Research Cluster University of Campinas Campinas São Paulo Brazil

## Abstract

**Introduction:**

While severe acute respiratory syndrome coronavirus 2 (SARS‐CoV‐2) has been extensively studied in the context of pulmonary disease, its impact on metabolic organs remains poorly understood, particularly across experimental models.

**Methods and Results:**

In this study, we investigated how infection with the SARS‐CoV‐2 B.1 and P.1 variants affects metabolic tissues in K18‐hACE2 transgenic mice. Both viral variants caused significant body weight loss and clinical deterioration as early as 3 days postinfection, with the P.1 variant leading to higher lethality than B.1. Notably, this increased disease severity was not associated with elevated viral loads in the lungs. Viral RNA was also detected in both the liver and adipose tissues, indicating that these metabolic organs are permissive to infection in this model. Histological analysis revealed disrupted hepatic parenchymal organization in P.1‐infected mice, even in the absence of substantial immune cell infiltration. Despite this, liver expression of pro‐inflammatory cytokines was significantly elevated, particularly following P.1 infection. These changes were not accompanied by liver fibrosis; however, tissue disorganization was confirmed using two‐photon excitation fluorescence imaging. In parallel, systemic metabolic alterations were observed, including increased ketogenesis, reduced adipose tissue mass, hepatic glycogen depletion, and alterations in glucose homeostasis. These metabolic disturbances likely contributed to the observed weight loss and reflect a host response to viral‐induced energy stress.

**Conclusion:**

Together, these findings demonstrate that SARS‐CoV‐2 infects metabolic tissues beyond the lungs and that the P.1 variant induces exacerbated hepatic stress and more severe disease outcomes in this experimental model.

## 1 Introduction

The Coronavirus Disease 2019 (COVID‐19) pandemic, caused by SARS‐CoV‐2, had profound global consequences, extending beyond public health into socioeconomic stability, scientific research, education, mental health, and cultural practices. In response, the scientific community mobilized rapidly to develop vaccines, identify therapeutic targets, and advance treatment strategies. Despite these efforts, the emergence of new viral variants continues to challenge disease management and raise questions regarding their transmissibility, immune evasion, pathogenicity, and response to existing therapies. Since the emergence of the ancestral Brazilian strain B.1, multiple SARS‐CoV‐2 variants have evolved, challenging our understanding of the viral biology and therapeutic efficacy. Among them, the P.1 variant, now designated “Gamma,” was first identified in December 2020 in Manaus, Brazil, and rapidly spread throughout the Amazonas region by January 2021. The P.1 variant carries three key mutations in the receptor‐binding domain (K417T, N501Y, and E484K) that confer increased binding affinity to the ACE2 receptor and enhanced immune evasion [1]. These features led the World Health Organization to classify it as a Variant of Concern due to its elevated transmissibility, virulence, reduced neutralization by antibodies, and diminished responsiveness to vaccines and therapeutics [2, 3, 4]. Although precise estimates of mortality directly attributable to this variant are hindered by limited genomic surveillance, it is widely recognized that P.1 contributed to a sharp increase in COVID‐19‐related hospitalizations and deaths in Brazil during the early months of 2021, particularly among younger individuals without pre‐existing conditions [5, 6]. During this period, the variant was implicated in higher mortality compared to previous lineages, highlighting the urgent need to understand its biological behavior and clinical implications [1].

A major early barrier in studying SARS‐CoV‐2 pathogenesis was the lack of an animal model that accurately mirrored the systemic effects of the virus in humans [7]. While standard laboratory mice are resistant to SARS‐CoV‐2 infection due to poor binding of the virus to the murine ACE2 receptor, this limitation was partially overcome by the development of the K18‐hACE2 transgenic mouse. This model expresses the human ACE2 receptor under the control of the epithelial‐specific keratin‐18 promoter, rendering mice susceptible to infection via the same cellular entry mechanism used in humans [8]. Upon intranasal inoculation, K18‐hACE2 mice develop a severe, often fatal disease that recapitulates several features of human COVID‐19, including pulmonary pathology, systemic viral dissemination, and multiorgan involvement [9]. The model has since become a cornerstone in COVID‐19 research, contributing to the understanding of viral spread, immune responses, and therapeutic efficacy [10, 11, 12, 13, 14].

The K18‐hACE2 mouse is permissive to infection by multiple SARS‐CoV‐2 variants, including the original Wuhan strain and subsequent Alpha, Beta, Delta, and Omicron variants. Studies have shown that infection with these variants reliably reproduces many aspects of the respiratory disease observed in humans [9, 15, 16]. Notably, recent findings demonstrate that infection with the Gamma variant in this model results in more severe disease outcomes than infection with either the original or Delta strains [17, 18]. Despite these advances, relatively little is known about how SARS‐CoV‐2, particularly the P.1 variant, affects host metabolic function in the K18‐hACE2 model. This knowledge gap is highly relevant, given that severe COVID‐19 has been strongly associated with pre‐existing metabolic disorders, such as obesity, type 2 diabetes, cardiovascular disease, and nonalcoholic fatty liver disease [19, 20]. Furthermore, accumulating evidence indicates that SARS‐CoV‐2 can directly infect metabolic tissues, including the liver and adipose tissue, in both humans and animal models, contributing to systemic metabolic disruption [21, 22, 23]. However, a direct comparison of the metabolic effects induced by the B.1 and P.1 variants in susceptible hosts is still lacking. To address this, the present study employed the K18‐hACE2 model to investigate and compare the impact of B.1 and P.1 SARS‐CoV‐2 variants on key metabolic organs. By focusing on liver and adipose tissue pathology, the goal of this work is to elucidate how SARS‐CoV‐2 infection disrupts systemic metabolic homeostasis and contributes to disease severity beyond the lungs.

## 2 Methods

### 2.1 Animals

Adult heterozygous female K18‐hACE2 transgenic mice were obtained from the Multidisciplinary Center for Biological Investigation (CEMIB), Campinas, São Paulo, Brazil. Mice were housed in standard filter‐top cages with ad libitum access to sterile water and food. All animal procedures were approved by the Ethics Committee on Animal Use of the University of Campinas (Protocol #5495‐1/2020).

### 2.2 SARS‐CoV‐2 Mouse Inoculation

The SARS‐CoV‐2 B strain (HIAE‐02‐SARS CoV‐2/SP02/human/2020/BRA; GenBank MT126808.1) was kindly provided by Prof. Dr. Edison Durigon (ICB‐USP, São Paulo, Brazil). Viral stocks were propagated in Vero cells (ATCC CCL81), harvested 2–3 days postinfection (dpi), and stored at −80°C. Viral titers were determined by plaque assay and RT‐qPCR, as previously described [24]. Virus production was carried out in a biosafety level 3 (BSL‐3) facility at the Laboratory of Emerging Viruses, Institute of Biology, UNICAMP, Campinas, Brazil. Mice were anesthetized using a combination of xylazine (20 mg/kg) and ketamine (100 mg/kg) prior to inoculation. For survival curve, mice were intranasally inoculated with 5 × 10^4^ plaque‐forming units (PFU) of SARS‐CoV‐2 in 40 μL. For all other experiments, including tissue collection at 5 dpi, mice received 1 × 10^4^ PFU. All infections were conducted in the BSL‐3 animal facility at Ribeirão Preto Medical School (FMRP), Ribeirão Preto, Brazil, by a designated researcher, ensuring that the principal experimental researcher remained blinded to group allocation during subsequent analysis. Following infection, animals were monitored daily for body weight changes and clinical signs of disease. Disease progression was assessed using a clinical scoring system that evaluated body weight loss, behavior, and respiratory symptoms, as described by Rodrigues et al. [17]. Clinical scores were calculated as the sum of six evaluated parameters per animal and are present as group means per day. At 5 dpi, mice were anesthetized with a ketamine–xylazine mixture (20 and 100 mg/kg, respectively). Blood samples were collected via the retrobulbar venous plexus prior to intracardially perfused with 20 mL of sterile saline solution. SARS‐CoV‐2 infection experiments were conducted independently on three occasions using K18‐hACE2 mice. The initial group size of at least 5 animals was chosen based on prior experience [17] showing that this number is sufficient to detect robust infection‐induced changes in tissue pathology and molecular readouts in this model. However, a subset reached humane endpoints prior to tissue collection. Consequently, longitudinal measurements such as body weight were recorded for all animals from the start of the experiments, whereas downstream analyses were performed only on animals that survived to the designated harvesting time point.

Mice were allocated into three groups: NI (noninfected), B.1 (infected with SARS‐CoV‐2 B.1 variant), and P.1 (infected with the P.1 variant). Because P.1‐infected mice exhibited greater disease severity, reduced survival in this group resulted in smaller sample sizes for certain analyses. The exact sample size (*n*) for each experiment is indicated in the corresponding figure legends. For the tissues analyzed in this study, the final numbers were as follows: liver samples included *n* = 5 (B.1‐infected, 5 dpi), *n* = 3 (P.1‐infected, 5 dpi), and *n* = 4 (noninfected controls). Adipose tissue samples included *n* = 3 (B.1‐infected, 5 dpi), *n* = 3 (P.1‐infected, 5 dpi), and *n* = 4 (noninfected controls). All animals were euthanized at 5 dpi, and tissues were collected for downstream analyses.

### 2.3 RNA Extraction and Viral Load Quantification

Liver, perigonadal, and subcutaneous fat depots were weighed and homogenized in 1 mL Hank's Balanced Salt solution (HBSS) supplemented with 1% penicillin–streptomycin and zirconia beads using a MagNA Lyser homogenizer (Roche Life Science, Mannheim, Germany). Homogenates were clarified by centrifugation and used for RNA extraction with the Quick‐RNA Viral Kit (Zymo Research, Irvine, CA, USA), following the manufacturer's protocol. RNA concentrations were determined using the NanoDrop 2000 spectrophotometer. Viral RNA quantification was performed as previously described by Rodrigues et al. [17]. Negative samples and a standard curve generated from serial 10‐fold dilutions of virus stock with known titers were included in each run. Viral loads were expressed as log_10_ viral RNA copies per gram of tissue or per milliliter after normalization.

### 2.4 Quantitative Real‐Time PCR Analysis

For genes other than SARS‐CoV‐2, total RNA was extracted using TRIzol reagent following the manufacturer's protocol (Sigma‐Merck). RNA quantification was performed as previously described, and 50 ng of RNA was used for reverse transcription using the High‐Capacity RNA‐to‐cDNA Kit (Thermo Fisher) according to the manufacturer's instructions. Quantitative real‐time PCR (qRT‐PCR) was carried out using Sybr Green Master mix (Applied Biosystems, Waltham, MA, USA) on a BIO‐RAD CFX394 Touch Real‐Time PCR Detection System. Relative gene expression levels were calculated using the 2^−ΔΔCt^ method, with 18S rRNA used as the endogenous control. Primer sequences were either obtained from previously published studies or designed using Primer‐BLAST and validated for specificity and efficiency prior to use (Table 1).

**Table 1:** Sequences of primers used for qRT‐PCR.

Gene	Forward primer	Reverse primer
Ldha	5′‐CATTGTCAAGTACAGTCCACA‐3′	5′‐TTCCAATTACTCGGTTTTTGG‐3′
Bnip3	5′‐TAAGGTTACCCACGAACCCC‐3′	5′‐GGTGCCTGCAACAAAACTGA‐3′
Gsdmd	5′‐GCGATCTCATTCCGGTGGACAC‐3′	5′‐TTCCCATCGACGACATCAGAGAC‐3′
Stat1	5′‐GCTGCCTATGATGTCTCGTTT‐3′	5′‐TGCTTTTCCGTATGTTGTGCT‐3′
Isg15	5′‐CATCCTGGTGAGGAACGAAAGG‐3′	5′‐CTCAGCCAGAACTGGTCTTCGT‐3′
Serping1	5′‐AGAGAGCTTCCCTGAAAGAGATG‐3′	5′‐TGAGGAGGCTGGCAATGCTGA‐3′
Mcp1r	5′‐AGCTGTAGTTTTTGTCACCAAGC‐3′	5′‐TGTCTGGACCCATTCCTTCTTG‐3′
Mcp1	5′‐AGCTGTAGTTTTTGTCACCAAGC‐3′	5′‐TGTCTGGACCCATTCCTTCTTG‐3′
Csf1r	5′‐CAGTTCAGAGTGATGTGTGGTC‐3′	5′‐CTTGTTGTTCACTAGGATGCCG‐3′
Csf1	5′‐AGTATTGCCAAGGAGGTGTCAG‐3′	5′‐ATCTGGCATGAAGTCTCCATTT‐3′
Il1b	5′‐GGCAGCTACCTGTGTCTTTCCC‐3′	5′‐ATATGGGTCCGACAGCACGAG‐3′
Il6	5′‐CACAGAGGATACCACTCCCAACA‐3′	5′‐TCCACGATTTCCCAGAGAACA‐3′
Tnfa	5′‐AGATAGCAAATCGGCTGACG‐3′	5′‐ACGGCATGGATCTCAAAGAC‐3′
Ifna	5′‐CCTGAGAGAGAAGAAACACAGCC‐3′	5′‐TCTGCTCTGACCACYTCCCAG‐3′
Ifny	5′‐GCTGATCCTTTGGACCCTCT‐3′	5′‐AGACTGCAAAGCCAAGATG‐3′
Rorc	5′‐GACCCACACCTCACAAATTGA‐3′	5′‐AGTAGGCCACATTACACTGCT‐3′
Glut1	5′‐GCTGTGCTTATGGGCTTCTC‐3′	5′‐CACATACATGGGCACAAAGC‐3′
Pfkfb3	5′‐GTCGCCGAATACAGCTACGA‐3′	5′‐CCCACAGGATCTGGGCAAC‐3′
Acad	5′‐ATGGGAGAAGCAGGCAAACA‐3′	5′‐ACTCTGGGTGGACAATCCCT‐3′
Fasn	5′‐GCATAACGGTCTCTGGTGGT‐3′	5′‐CGAACTTTTCCAAGGTGGGG‐3′
Ndfus8	5′‐CTAGCAGAAACAAACCGGGC‐3′	5′‐CCGGCTGCGTATTCTACGTT‐3′
Sdha	5′‐GGAACACTCCAAAAACAGACCT‐3′	5′‐CCACCACTGGGTATTGAGTAGAA‐3′
Uqcrc2	5′‐AAAGTTGCCCCGAAGGTTAAA‐3′	5′‐GAGCATAGTTTTCCAGAGAAGCA‐3′
Cox6a1	5′‐TCAACGTGTTCCTCAAGTCGC‐3′	5′‐AGGGTATGGTTACCGTCTCCC‐3′
Cox15	5′‐CTCTGTGGCTTAGTCTGTTTCC‐3′	5′‐CGAGGGATGTCATAGGACTCTG‐3′
Cox4a1	5′‐GCGGTACAACTGAACTTTCTC‐3′	5′‐AGTGTTGTGAAGAGTGAAGAC‐3′
Atp5a1	5′‐TCTCCATGCCTCTAACACTCG‐3′	5′‐CCAGGTCAACAGACGTGTCAG‐3′
Mfn1	5′‐CAGAGAAGAGGGTTTATTCA‐3′	5′‐ACTCATCAACCAAAACAGAT‐3′
Mfn2	5′‐TGAATGTTGTGTTCTTTCTG‐3′	5′‐AAGTGCTCTCTGCTAAATGT‐3′
Mff	5′‐AGCTGCCGCCACTTCTAATC‐3′	5′‐TGCATCTACCACAGTCATGTCA‐3′
Drp1	5′‐GACCCATTCATTTGTCACG‐3′	5′‐CGGTTCCCTAAACTTCACGA‐3′

### 2.5 Biochemical Parameter Analysis

The activity of alanine aminotransferase (ALT) and aspartate aminotransferase (AST) was measured using Labtest kits (Cat. 53 and 52), according to the manufacturer's instructions. Blood glucose and ketone levels were measured using peripheral blood samples collected from the tail vein or prior to titers intracardiac perfusion, with a glucometer (FreeStyle Optium Neo, Abbott).

### 2.6 Histological Analysis

After transcardiac perfusion, perigonadal, subcutaneous and liver were harvested and fixed in 4% paraformaldehyde for 72 h at 4°C. After fixation, samples were washed in saline solution and stored in 70% ethanol. Then, tissues were embedded in paraffin and sectioned transversely into 5 μm slices on a microtome. Subsequent sections were stained for either hematoxylin and eosin (H&E) and periodic acid‐Schiff (PAS) to detect glycogen. After deparaffinization and rehydration, liver sections were treated with 1% cold periodic acid for 30 min, which oxidizes glycogen to form aldehyde groups. These aldehydes reacted with Schiff's reagent in a dark chamber for 30 min, producing a magenta‐violet coloration. Thereafter, hematoxylin counterstain was applied to provide contrast.

### 2.7 RNAscope In Situ Hybridization for SARS‐CoV‐2 Detection

The detection of SARS‐CoV‐2 RNA in lung sections was also performed using the RNAscope in situ hybridization (ISH) assay (Advanced Cell Diagnostics, Newark, CA, USA) following the manufacturer's instructions (Reference 322452). Briefly, paraffin‐embedded lung tissue sections (5 µm thick) were deparaffinized in xylene, rehydrated through a graded ethanol series, and treated with hydrogen peroxide for 10 min at room temperature to block endogenous peroxidase activity. Tissue sections were then subjected to heat‐induced target retrieval in RNAscope Target Retrieval Reagent at 98°C–100°C for 15 min, followed by protease digestion using RNAscope Protease Plus at 40°C for 30 min in a HybEZ oven (Advanced Cell Diagnostics). After pretreatment, the tissues were hybridized with a target‐specific probe for SARS‐CoV‐2 RNA (V‐nCoV2019‐S probe, Cat No. 848561, ACD) for 2 h at 40°C.

Signal amplification was achieved through sequential hybridization steps using the RNAscope 2.5 HD Detection Kit, according to the manufacturer's protocol. Sections were counterstained with DAPI and mounted with an appropriate medium. Positive staining was identified by distinct dark red puncta within the cytoplasm of infected cells or in the lung parenchyma. A no‐probe negative control and SARS‐CoV‐2‐infected tissues known to be positive served as internal controls to validate assay specificity.

### 2.8 Nonlinear TPEF and SHG Microscopy

Second harmonic generation (SHG) and two‐photon excitation fluorescence (TPEF) images of tissues infected with SARS‐CoV‐2 were acquired using an inverted confocal microscope Zeiss LSM 780‐NLO Axio Observer Z.1 (Carl Zeiss AG, Germany), equipped with a femtosecond pulsed laser (pulse duration: 100 fs, repetition rate: 80 MHz), model Chameleon Discovery NX (Coherent Inc., Santa Clara, California), housed at the National Institute of Science and Technology on Photonics Applied to Cell Biology (INFABIC), University of Campinas, São Paulo, Brazil. The laser beam was directed into the microscope via a set of mirrors, passing through a half‐wave plate combined with a polarizer for laser power control, a beam collimating telescope (T), and a quarter‐wave plate (*λ*/4). The beam was then focused onto the sample using an oil immersion objective (EC Plan‐Neofluar 40×/1.3, Carl Zeiss AG, Germany). All SHG images were acquired using an excitation wavelength of 940 nm with an average power of 20 mW at the sample plane. The SHG signal was collected in the forward (transmitted) direction using a condenser with 0.55 NA and a working distance of 26 mm (Carl Zeiss AG, Germany), and detected by a nondescanned detector (T) after passing through a filter cube containing an SP/485 filter. Meanwhile, the TPEF signal was collected in the backward (epi‐detection) direction by a nondescanned detector (R), following passage through a filter cube containing a dichroic mirror (Carl Zeiss, 1512‐461) and an LP/490 filter. After excitation, the laser beam was blocked in both the transmitted and reflected detection paths using an SP/690 optical filter. The collimating telescope was used to match the beam diameter to the back aperture of the objective, and the quarter‐wave plate (*λ*/4) was employed to uniformly excite collagen fibers in all orientations.

### 2.9 Microscopy Image Analysis

All microscopy image analyses were performed using FIJI/ImageJ software. The quantification of glycogen content in liver sections stained with PAS was conducted using Colour Deconvolution plugin with the H PAS vector to separate the PAS signal from the hematoxylin counterstain. The PAS‐specific channel was selected, and a threshold was applied to isolate the positively stained areas. These areas were converted into binary masks, which were slightly enlarged to ensure full coverage of the stained regions. Regions of interest (ROIs) were added to the ROI Manager and used to measure the integrated density (IntDen), which served as a quantitative indicator of glycogen accumulation in the liver tissue. Collagen deposition was quantified on SHG images. The IntDen of the collagen signal was measured and subsequently normalized by the number and diameter of blood vessels present in each image. This normalization accounted for tissue vascularization variability and improved the accuracy of the collagen quantification.

Tissue organization was evaluated in TPEF images by measuring the orientation of structural features using the Directionality plugin in FIJI/ImageJ. This analysis provides an estimate of how aligned or heterogeneous the tissue structure is. The goodness‐of‐fit value (*R* ^2^) was used as a measure of alignment: higher *R* ^2^ values indicate that structures are more uniformly oriented, whereas lower *R* ^2^ values reflect a more complex and heterogeneous tissue architecture. Importantly, increased alignment (higher *R* ^2^) does not necessarily indicate healthy organization, but may instead reflect tissue remodeling or loss of normal parenchymal complexity.

### 2.10 Statistical Analysis

Statistical analyses were performed using GraphPad Prism 8.0 software (San Diego, CA, USA). Unless otherwise specified in the figure captions, data are presented as mean ± standard error of the mean (SEM). Comparisons between two groups were performed using unpaired Student's *t*‐test. For comparisons involving more than two groups, one‐way ANOVA followed by Tukey's post hoc test was applied. A *p* < 0.05 was considered statistically significant. Data distribution was assessed for normality. Given the small sample size and the robustness of ANOVA to moderate deviations from normality, parametric tests were applied.

## 3 Results

### 3.1 Metabolic Organs Are Equally Vulnerable to SARS‐CoV‐2 B.1 and P.1 Infection in K18‐hACE2 Mice

**Figure 1:**
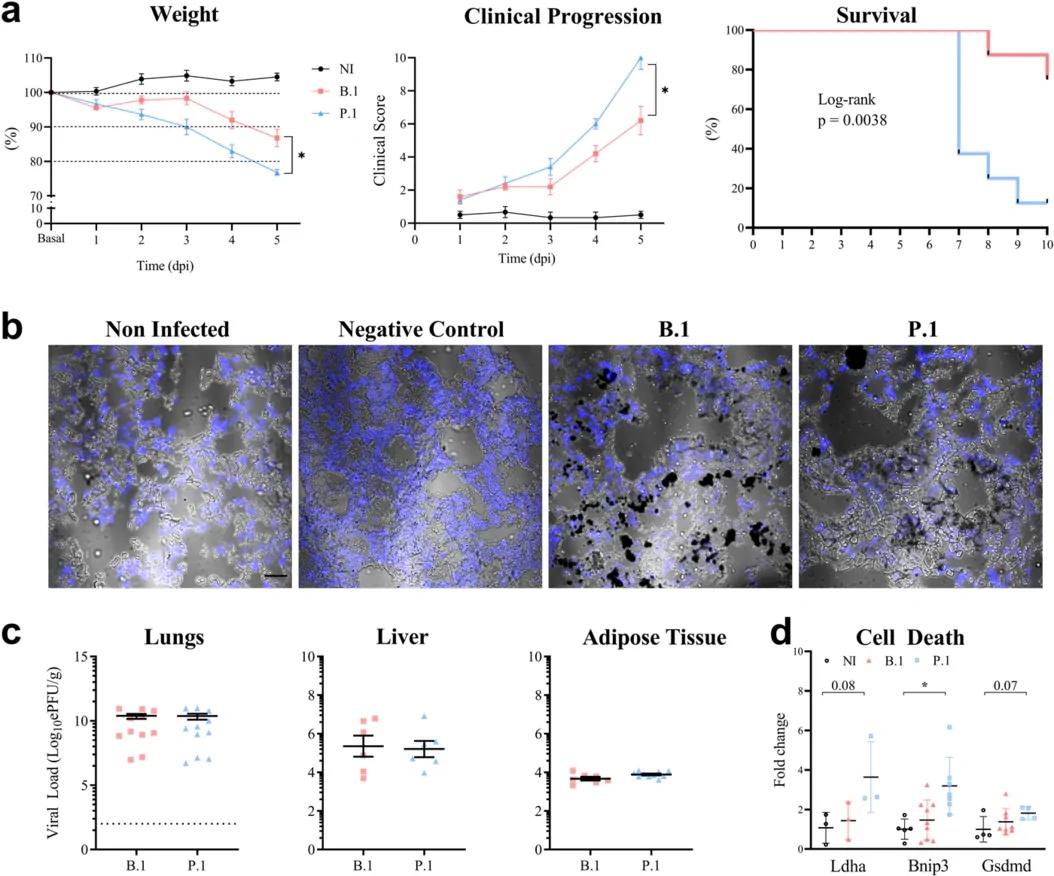
Infection of K18‐hACE2 mice with SARS‐CoV‐2 variants. (a) Body weight, clinical score, and survival of female K18‐hACE2 mice infected with either B.1 or P.1 SARS‐CoV‐2 variants, monitored over the course of infection. Body weight is expressed as a percentage of initial weight (set at 100%; *n* = 12–15), with linear regression *p* < 0.0001. Clinical scores represent the daily mean per group (*n* = 12–15), based on the scoring criteria previously published [17]; statistical analysis by Log‐rank (Mantel–Cox) test, *p* = 0.0038. Survival is shown as the percentage of living animals (*n* = 6), with 100% as baseline; linear regression *p* = 0.0012. (b) Representative RNAscope images of lung sections showing S protein staining. Nuclei are labeled with DAPI (blue), and SARS‐CoV‐2 S protein is visualized in dark violet. A negative control was performed using a negative probe on B.1‐infected samples, confirming the specificity of the staining. Scale bar: 20 µm. (c) Viral load in lung tissue quantified by RT‐qPCR and normalized to tissue weight (*n* = 6–11 per group). (d) Expression of cell death‐related genes assessed by RT‐qPCR (*n* = 5). *p = 0.01, one‐way ANOVA.

**Figure 2:**
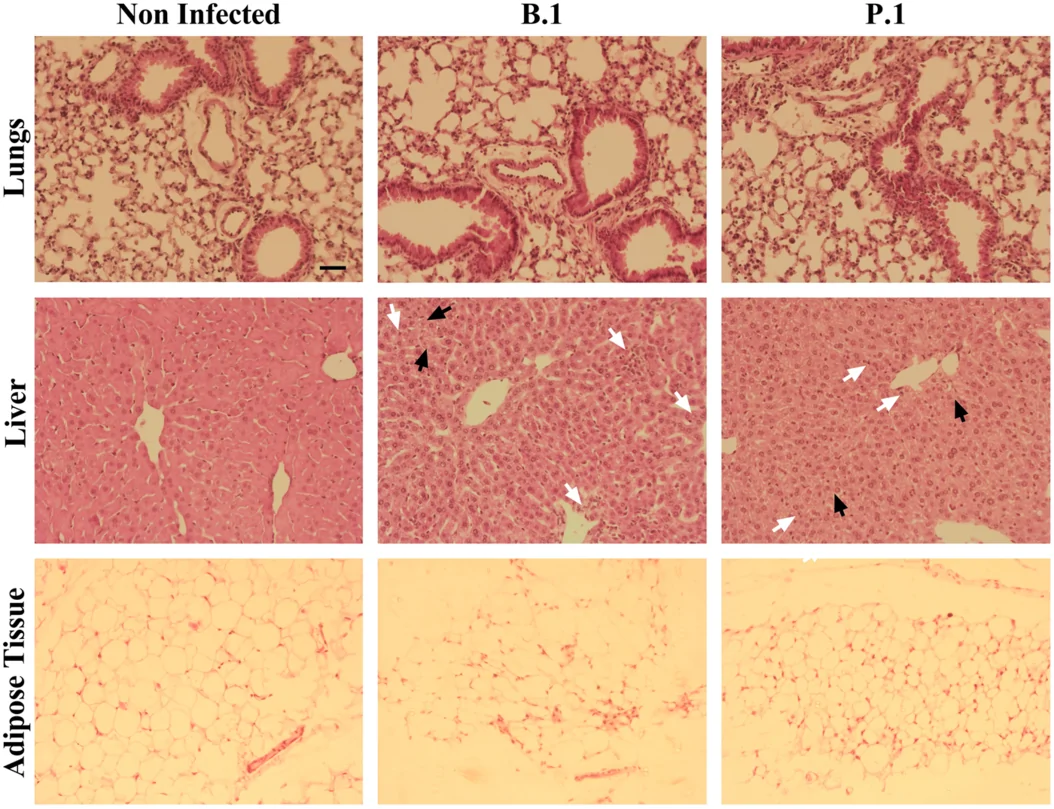
B.1 and P.1 SARS‐CoV‐2 infection does not induce overt immune cell infiltration in liver and adipose tissues. Hematoxylin and eosin (H&E) staining of liver and adipose tissues from K18‐hACE2 mice infected with SARS‐CoV‐2 B.1 and P.1 variants. Black arrows indicate the presence of cytoplasmic vacuoles in hepatocytes; white arrows indicate hepatocytes undergoing apoptosis or necrosis. No prominent immune cell infiltration was observed. Scale bar: 50 µm. Analysis was performed with *n* = 3 per group.

To investigate the impact of SARS‐CoV‐2 infection on metabolic tissues, female K18‐hACE2 transgenic mice were intranasally infected with either the B.1 or P.1 SARS‐CoV‐2 variant. Both viral strains induced approximately 20% body weight loss and worsened clinical score by 3 dpi, resulting in reduced survival. Infection with the P.1 variant resulted in greater disease severity and lethality, with only 12.5% survival compared to 75% of B.1‐infected mice (Figure 1a). This difference in lethality was not attributed to increased viral burden, as both variants exhibited comparable RNA levels across tissues. In the lungs, viral load was similar between B.1 (2.48 × 10^10^ ± 1.05 × 10^10^) and P.1 (2.42 × 10^10^ ± 1.16 × 10^10^), as assessed by RNAscope and qPCR (Figure 1b,c). Likewise, no differences were observed in ACE2 expression between groups (data not shown). Beyond the lungs, viral RNA from both variants was also detected in metabolic organs, including the liver and adipose tissue, with comparable levels between strains. In the liver, viral load was 5.36 × 10^10^ ± 0.55 × 10^10^ (B.1) and 5.21 × 10^10^ ± 0.42 × 10^10^ (P.1), while in adipose tissue it was 3.67 × 10^10^ ± 0.09 × 10^10^ (B.1) and 3.89 × 10^10^ ± 0.05 × 10^10^ (P.1) (Figure 1c). These findings indicate that liver and adipose tissues are permissive to SARS‐CoV‐2 infection, and that the increased lethality associated with the P.1 variant is not driven by higher viral loads. We next asked whether variant‐specific differences in host responses within these tissues could account for the observed differences in disease severity. In addition, only the P.1 infection led to increased hepatic expression of cell death pathway‐related genes, including *Ldha*, *Bnip3*, and *Gsdmd* (Figure 1d), an effect not observed in adipose tissue (data not shown). Despite the presence of viral RNA in both liver and adipose tissues, histological analysis revealed no evidence of increased immune cell infiltration in these organs (Figure 2). Additionally, H&E staining revealed the presence of vacuoles in hepatocytes, along with morphological features of necrosis and apoptosis following the infection with both viral variants. Collectively, these findings demonstrate that both B.1 and P.1 SARS‐CoV‐2 variants can infect metabolic organs in K18‐hACE2 mice, with P.1 infection being specifically associated with enhanced hepatic stress and greater disease severity.

### 3.2 The P.1 Variant Disrupts Liver Parenchyma Isotropy and Triggers Pro‐Inflammatory Response

**Figure 3:**
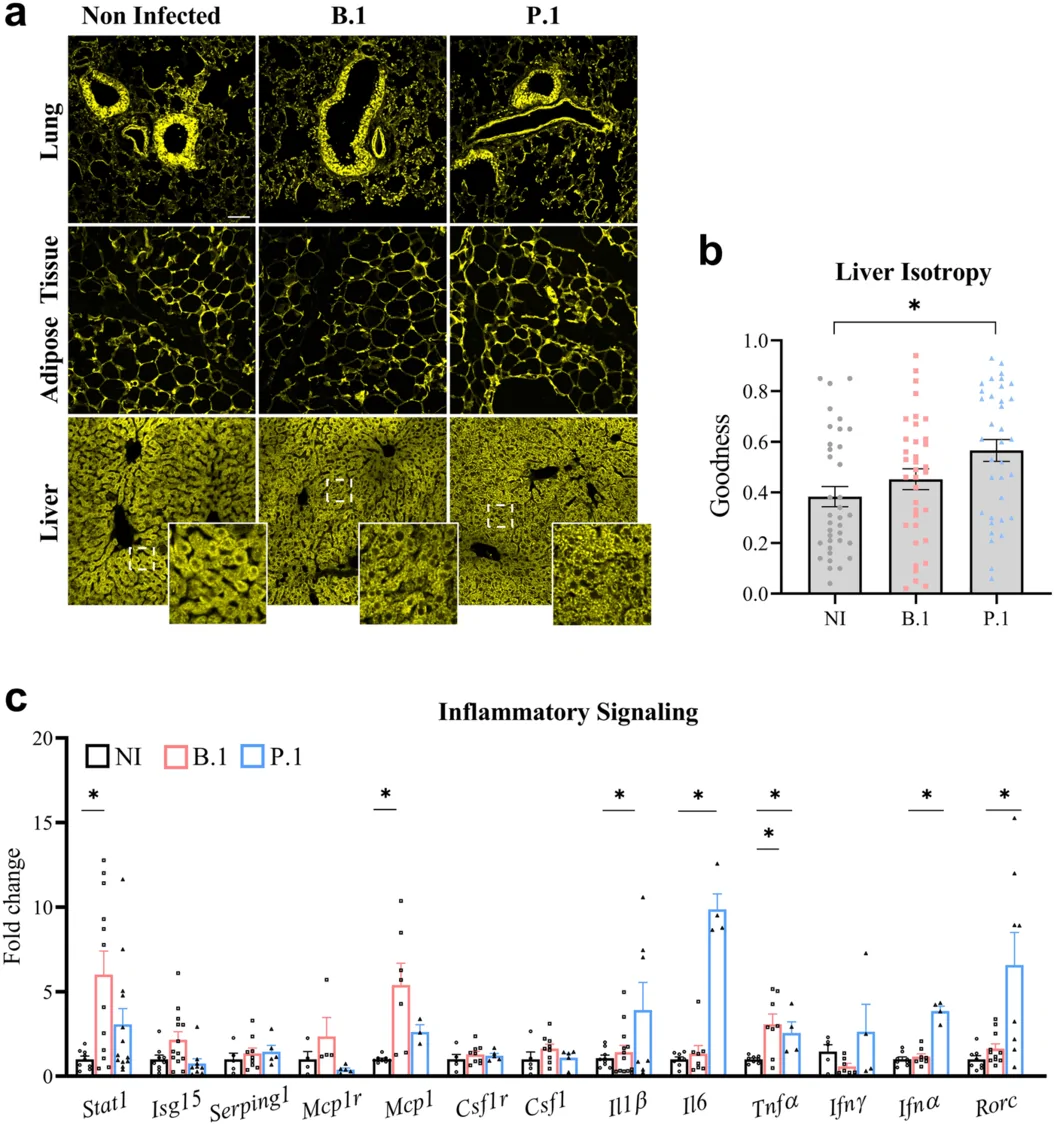
The P.1 variant disrupts liver parenchyma organization, and both B.1 and P.1 variants trigger an inflammatory response in the liver. (a) TPEF imaging reveals the structural integrity of lung, adipose, and liver tissues following infection with SARS‐CoV‐2 B.1 and P.1 variants. Insets at the bottom right of each image show magnified views of the regions highlighted with yellow dashed squares. Scale bar: 50 µm. (b) Quantification of liver parenchyma isotropy demonstrates significantly increased disruption in P.1‐infected mice compared to noninfected (NI) controls (*n* = 4 per group; 9 images per animal); unpaired Student's *t*‐test, *p* = 0.0028. (c) RT‐qPCR analysis of inflammatory gene expression in liver tissue (*n* = 4 per group), presented as fold change relative to NI controls. Statistical analysis was performed using one‐way ANOVA followed by multiple comparisons. *p < 0.05. The following comparisons were significant: Stat1 (NI vs. B.1, *p* < 0.0001), Mcp1 (NI vs. B.1, *p* < 0.0001), Il1b (NI vs. B.1, *p* < 0.0001), Il6 (NI vs. P.1, *p* < 0.0001), Rorc (NI vs. P.1, *p* < 0.0001), and Rorc (B.1 vs. P.1, *p* = 0.0002). Additional pairwise comparisons using unpaired Student's *t*‐test showed: Tnfα (NI vs. B.1, *p* = 0.0046; NI vs. P.1, *p* = 0.0071) and Ifnα (NI vs. B.1, *p* = 0.04).

To assess morphological alterations in metabolic organs following SARS‐CoV‐2 infection, we analyzed the parenchyma of the lungs, liver, and adipose tissue using TPEF microscopy. This nonlinear imaging technique employs the simultaneous absorption of two long‐wavelength photons to excite endogenous fluorophores, enabling the visualization of structural and biochemical features in tissues without staining. TPEF allows detection of intrinsic fluorescence from molecules such as elastin, NAD(P)H, FAD, and lipofuscin, offering insights into tissue architecture and metabolism. TPEF optical sectioning revealed structural differences in the liver parenchyma between infected and noninfected mice, while no alterations were detected in lung or adipose tissue (Figure 3a). The hepatic parenchyma normally consists of two‐cell‐thick hepatocyte cords separated by irregular sinusoids, which dynamically vary in diameter in response to hemodynamics changes [25]. Notably, infection with P.1 variant resulted in a marked change in liver isotropy, with increased hepatocyte alignment (anisotropy) and the appearance of intense fluorescent foci (Figure 3a). Importantly, increased alignment does not necessarily correspond to preserved tissue organization, in our context, higher *R* ^2^ values observed upon SARS‐CoV‐2 infection likely reflect pathological remodeling and loss of normal parenchymal complexity, such as sinusoidal narrowing and reorganization of hepatocyte cords, as confirmed by quantitative analysis of fluorescence directionality (goodness‐of‐fit, Figure 3b). To investigate whether these structural alterations were associated with inflammation, we evaluated the expression of immune cell markers and cytokines. Although no histological signs of immune infiltration were observed, gene expression analysis showed that both B.1 and P.1 variants triggered the expression of pro‐inflammatory mediators. While both strains increased *Tnfα* expression, the P.1 variant significantly upregulated *Il1β*, *Il6* and the transcription factor *Rorc*, which is associated with Th17 immune responses (Figure 3c). Additionally, both variants induced *Mcp1*, a chemokine that plays a crucial role in immune recruitment of monocytes, and *Stat1* expression, a critical transcription factor that mediates the cellular response to interferons by activating the expression of interferon‐stimulated genes, which are essential for antiviral defense and myeloid cell activation. Supporting this, we observed increased expression of *Ifnɑ*, specifically in P.1‐infected livers, indicating a robust type I interferon response. In summary, the P.1 variant elicits more pronounced disruption of liver architecture and induces stronger inflammatory gene expression compared to B.1. These alterations may contribute to the increased severity and lethality observed in P.1‐infected K18‐hACE2 mice.

### 3.3 SARS‐CoV‐2 Infection Does Not Induce Liver Fibrosis

**Figure 4:**
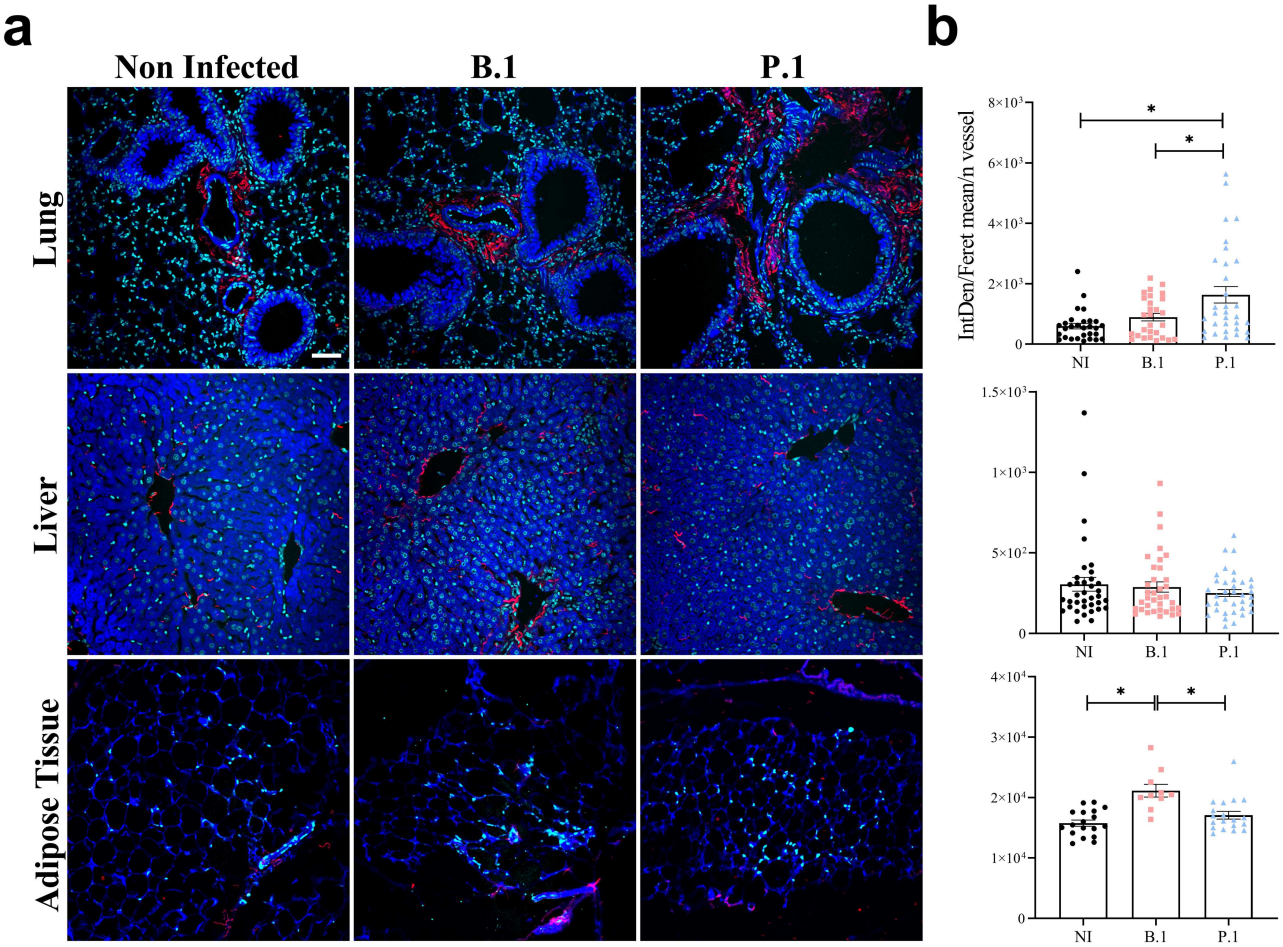
SARS‐CoV‐2 infection induces collagen deposition in lungs and adipose tissues. (a) Representative merged images of two‐photon excitation fluorescence (TPEF, blue), second harmonic generation (SHG, red), and third harmonic generation (THG, cyan) showing collagen fiber distribution in lung (red), liver, and adipose tissues from mice infected with SARS‐CoV‐2 B.1 and P.1 variants. Scale bar: 50 µm. (b) Quantification of collagen fluorescence intensity (integrated density, IntDen), normalized by the number and diameter of blood vessels in each image. Data represent *n* = 3–4 mice per group, with 3–5 images per animal. *p < 0.05. Statistical analysis was performed using one‐way ANOVA followed by multiple comparisons. In the lung, significant differences were observed between NI and P.1 (*p* = 0.0005) and between B.1 and P.1 (*p* = 0.01). In adipose tissue, significant differences were found between NI and B.1 (*p* < 0.0001) and between B.1 and P.1 (*p* = 0.001).

Clinical studies have shown that more than half of COVID‐19 patients present with elevated liver fibrosis indices, such as the FIB‐4 score, which are positively correlated with higher viral loads and increased IL‐6 levels [24, 26, 27, 28, 29]. Given the altered inflammatory gene expression and changes in liver parenchyma observed in K18‐hACE2 model, we next assessed whether SARS‐CoV‐2 infection leads to hepatic fibrosis in these mice. To quantify collagen deposition, we employed SHG microscopy, a label‐free, nonlinear optical imaging technique that specifically highlights noncentrosymmetric structures such as fibrillar collagen. Quantitative analysis of SHG intensity revealed no signs of liver fibrosis following infection with either the B.1 or P.1 SARS‐CoV‐2 variants (Figure 4a), with comparable collagen signal across groups (NI: 305.3 ± 42.58; B.1: 288.5 ± 32.12; P.1: 251.1 ± 21.53). In contrast, the P.1 variant did induce collagen accumulation in the lungs, as reflected by increased SHG intensity (NI: 597.1 ± 92.24; B.1: 892.3 ± 124.9; P.1: 1634 ± 271.9), consistent with reports of pulmonary fibrosis in COVID‐19 patients [30, 31, 32] and in other models of viral respiratory disease [33]. In adipose tissue, collagen levels were elevated in B.1‐infected mice (NI: 15,756 ± 510.7; B.1: 21,121 ± 1061), whereas P.1‐infected animals showed levels comparable to noninfected controls (P.1: 17,062 ± 644.2) (Figure 4b). These findings suggest that, while SARS‐CoV‐2 can impact tissue structure and immune signaling in the liver, it does not necessarily lead to fibrogenesis in this organ within the experimental timeframe of the model used in this study.

### 3.4 SARS‐CoV‐2 Infection Disrupts Energy Metabolism Pathways

**Figure 5:**
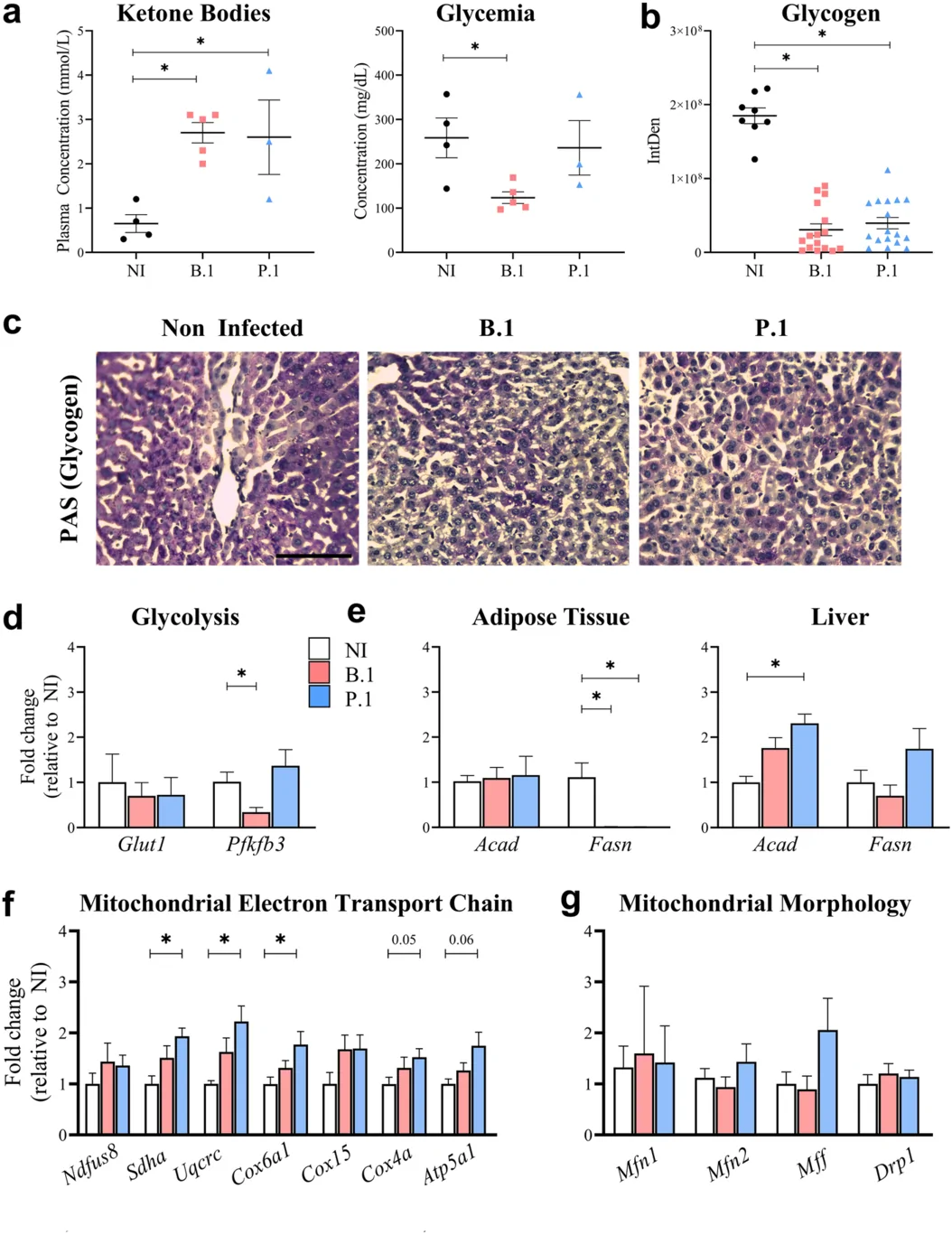
SARS‐CoV‐2 variants alter the metabolic state of infected mice. (a) Blood ketone and glucose levels measured at 5 dpi (*n* = 3–5 per group). (b) Quantification of liver glycogen content based on integrated density of violet PAS‐positive staining, corresponding to the representative images shown in (c) (*n* = 3 animals per group; 4 images per animal). (c) Representative brightfield images of periodic acid–Schiff (PAS) staining in liver sections. Scale bar: 50 µm. (d–g) RT‐qPCR analysis of glycolysis‐, lipid metabolism‐, mitochondrial electron transport chain‐, and mitochondrial morphology‐related gene expression in liver tissue or adipose tissue (where indicated) (*n* = 4 per group). Data are presented as fold change relative to NI mice. *p < 0.05. Statistical analysis was performed using unpaired Student's *t*‐test. Significant differences were observed as follows: ketone bodies (NI vs. B.1, *p* = 0.0003; NI vs. P.1, *p* = 0.04); glycemia (NI vs. B.1, *p* = 0.01); glycogen content (NI vs. B.1, *p* < 0.0001; NI vs. P.1, *p* < 0.0001); glycolysis: Pfkfb3 (NI vs. B.1, *p* = 0.02); adipose tissue lipid metabolism: Fasn (NI vs. B.1, *p* = 0.01; NI vs. P.1, *p* = 0.01); liver lipid metabolism: Acad (NI vs. P.1, *p* = 0.0008); mitochondrial electron transport chain: Sdha (NI vs. P.1, *p* = 0.0024), Uqcrc (NI vs. P.1, *p* = 0.01), and Cox6a1 (NI vs. P.1, *p* = 0.04).

Metabolic disturbances are common in COVID‐19 and include hyperglycemia, increased lipolysis, and ketosis [34, 35, 36]. These alterations result from a combination of systemic inflammation, ACE2 downregulation, and direct viral injury to pancreatic *β*‐cells. Additionally, SARS‐CoV‐2 has been shown to promote glycolysis‐dependent cytokine production in immune cells and induce mitochondrial dysfunction across various tissues [37, 38, 39, 40]. To determine whether the observed molecular and tissue alterations translate into systemic metabolic dysfunction, we assessed glycemia and ketonemia. In K18‐hACE2 mice, both SARS‐CoV‐2 variants significantly increased circulating ketone bodies levels (Figure 5a), suggesting enhanced lipid oxidation. However, glycemia was not elevated following P.1 infection and was even reduced in mice infected with the B.1 strain (Figure 5a). Correspondingly, hepatic glycogen stores were markedly depleted in both infection groups (Figure 5b,c), likely to reflect a compensatory effort by the liver to maintain blood glucose levels. To explore this further, we examined the expression of key glycolytic genes in the liver. Although *Glut1*, a major glucose transporter, showed no significant changes, expression of *Pfkfb3*, a critical regulator of glycolytic flux, was reduced following B.1 infection, suggesting impaired glycolysis (Figure 5d). These results underscore a systemic attempt to preserve glucose homeostasis under viral challenge.

**Figure 6:**
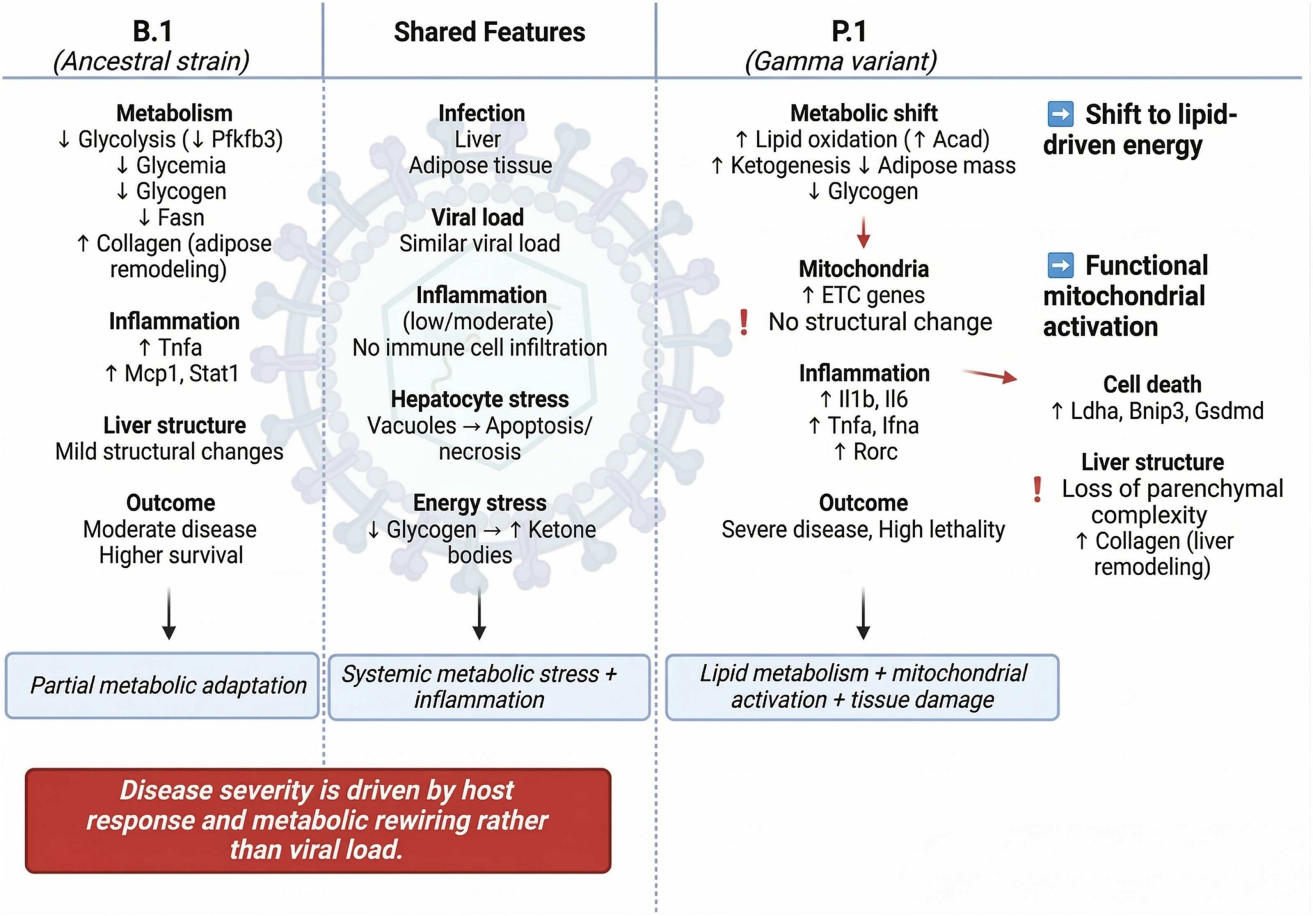
Variant‐specific metabolic and tissue remodeling responses to SARS‐CoV‐2 infection. Schematic representation summarizing the differential effects of B.1 and P.1 SARS‐CoV‐2 variants in K18‐hACE2 mice. Both variants infect metabolic organs, including the liver and adipose tissue, with comparable viral loads and in the absence of overt immune cell infiltration, leading to hepatocyte stress, glycogen depletion, and increased ketogenesis, indicative of systemic energy imbalance. In B.1‐infected mice, metabolic alterations are characterized by reduced glycolysis, decreased glycemia, and moderate inflammatory responses, accompanied by mild tissue remodeling and increased collagen deposition in adipose tissue. These changes are associated with a partial metabolic adaptation and moderate disease severity. In contrast, P.1 infection induces a pronounced metabolic shift toward lipid utilization, as evidenced by reduced adipose tissue mass, increased ketone bodies, and upregulation of fatty acid β‐oxidation genes. This is accompanied by increased expression of mitochondrial electron transport chain genes without detectable changes in mitochondrial morphology, suggesting functional mitochondrial activation rather than structural remodeling. P.1 infection also triggers stronger inflammatory responses, enhanced expression of cell death‐related genes, and marked disruption of liver architecture, including increased structural alignment and collagen deposition.

The reduction in glycolysis likely contributes to the metabolic shift toward lipid utilization observed in these mice. Indeed, adipose tissue mass was decreased postinfection (data not shown), which was accompanied by a significant downregulation of *Fasn*, a key enzyme involved in de novo lipogenesis (Figure 5e). Supporting the metabolic shift, hepatic expression of *Acad*, an enzyme involved in the initial steps of fatty acid *β*‐oxidation, was upregulated in P.1‐infected mice (Figure 5e), suggesting increased lipid catabolism in the liver of P.1‐infected mice. Since mitochondrial adaptations following SARS‐CoV‐2 infection have already been reported in both rodents and humans [39], we examined the expression of genes related to mitochondrial bioenergetics and morphology in the liver. Notably, P.1 infection led to the upregulation of several respiratory chain genes (Figure 5f), without detectable alterations in mitochondrial morphology (Figure 5g). Together, these findings demonstrate a preferential reliance on lipid metabolism during SARS‐CoV‐2 infection in K18‐hACE2 mice, particularly with the P.1 variant. This is supported by increased ketogenesis, reduced adipose tissue mass, glycogen depletion, and potentially suppression of glycolysis. These metabolic shifts may contribute to the observed weight loss in infected animals and reflect a systemic adaptation to viral‐induced energy stress (Figure 6).

## 4 Discussion

Here, we compared the effects of the B.1 (ancestral Brazilian strain) and P.1 (Gamma variant) SARS‐CoV‐2 lineages on metabolic organs, particularly the liver and adipose tissue of K18‐hACE2 mice. We identified strain‐specific differences in pathological and metabolic responses, highlighting the systemic nature of SARS‐CoV‐2 infection and its variant‐dependent impacts. Both B.1 and P.1 strains were capable of reaching and replicating in the liver and adipose tissues, as evidenced by detectable viral RNA. This occurred in the absence of large immune cell infiltration, suggesting that direct viral effects or low‐grade inflammation may contribute to local metabolic disruption. Notably, P.1 infection resulted in more severe clinical outcomes, including greater weight loss, worsened clinical score, and higher lethality, despite similar viral burdens compared to B.1 strain across tissues. These findings point to qualitative differences in host responses beyond viral load. Consistent with this interpretation, P.1 infection was associated with a heightened inflammatory and cell death signature, including increased expression of Il1b, Il6, Tnfα, Ifnα, and Rorc, suggesting an exacerbated and potentially maladaptive immune response. This was accompanied by marked disruption of liver parenchymal organization and enhanced collagen deposition, pointing to early tissue remodeling and fibrogenic processes. Such features resemble pathological responses described in severe and lethal COVID‐19 [8, 40], in which dysregulated inflammation and aberrant repair mechanisms, rather than viral burden alone, drive tissue damage and disease progression. In parallel, P.1 infection induced distinct metabolic alterations in the liver, which may reflect a compensatory response to elevated energetic demand and cellular stress. However, in the context of increased cell death and structural disorganization, this metabolic shift is likely insufficient to preserve tissue function, instead contributing to a state of metabolic imbalance.

Histological analysis revealed vacuolation or hydropic degeneration, necroptosis, and inflammatory foci in infected livers, and more extensive hepatic disorganization in P.1‐infected mice. These structural alterations were accompanied by transcriptional upregulation of inflammatory and cell‐death‐related genes. The upregulation of these genes, particularly following P.1 infection, suggests heightened hepatocyte stress and underlies pyroptotic and necroptotic pathways. Studies with other strains on post‐COVID‐19 sequelae have shown similar results, presence of steatosis‐like vacuoles and necroinflammation in the liver [41, 42, 43], suggesting a common characteristic of SARS‐CoV‐2 virus.

Although immune cell infiltration was limited in the transgenic mice and we did not observe AST/ALT alterations (data not shown), the gene expression signature pointed to an active tissue‐intrinsic inflammatory response. Complementing these findings, TPEF imaging revealed disrupted hepatic parenchymal organization in P.1‐infected animals, characterized by reduced sinusoidal spacing and the presence of eosinophilic body‐like structures, features indicative of altered microarchitecture, localized hypoxia, and early fibrotic‐like changes, as previously described by Stanciu et al. [44]. To investigate whether these structural disruptions were accompanied by fibrotic remodeling, we assessed collagen deposition using SHG imaging. We observed clear collagen deposition in the lungs of P.1‐infected mice, consistent with pulmonary fibrosis reported in COVID‐19 patients [45, 46]. Despite observing marked hepatic alterations, we did not detect clear evidence of fibrosis in the liver. One possible explanation is the presence of the dense Glisson's capsule in the surface of lobules, which may absorb the SHG signal and limit the visualization of deeper fibrotic changes within the hepatic parenchyma. Moreover, the development of fibrosis typically requires a prolonged disease course and may be influenced by host‐specific factors such as pre‐existing comorbidities, conditions not replicated in this acute infection model. Notably, findings from human autopsy studies have reported hepatocyte injury, endothelial infection, and signs of early‐stage fibrosis in COVID‐19 patients [47, 48, 49]. In this context, our murine data reflect several of these pathological features, particularly hepatic disorganization and inflammatory gene activation, even in the absence of overt immune cell infiltration. These similarities suggest that the K18‐hACE2 model, especially following P.1 infection, may serve as a useful tool for investigating the SARS‐CoV‐2–induced liver injury.

From a metabolic perspective, our findings support predictions from genome‐scale metabolic models identifying SARS‐CoV‐2 as an inducer of hepatocellular metabolic reprogramming, particularly favoring fatty acid oxidation [50]. We observed hepatic glycogen depletion, elevated blood ketone levels, and upregulation of mitochondrial oxidative phosphorylation (OXPHOS) genes, especially in P.1‐infected mice. This suggests an early metabolic adaptation toward lipid mobilization. Importantly, these changes appeared to occur without alterations in mitochondrial morphology, indicating a functional adaptation at the transcriptional level rather than structural shift. In this context, the upregulation of respiratory chain components may reflect an increased reliance on oxidative metabolism to meet elevated energetic demands during infection. Consistent with this interpretation, P.1‐infected mice exhibited features of enhanced lipid utilization, including increased expression of fatty acid oxidation genes (e.g., *Acad*), elevated ketone bodies, depletion of adipose tissue, and reduced hepatic glycogen stores. While B.1 infection appeared to potentially reduce glycolysis (e.g., via reduced *Pfkfb3* expression), P.1 appears to promote fatty acid catabolism and ketone body production, suggesting distinct metabolic strategies for energy compensation. These findings partially diverge from human autopsy data showing hepatic downregulation of OXPHOS genes and greater mitochondrial disruption [39]. This discrepancy may reflect differences between acute infection in animal models and later‐stage disease in human patients.

Conversely, adipose tissue also emerged as an extrapulmonary site of infection, with viral RNA and molecular alterations detectable following both strains. In human adipose studies, adipocytes and resident macrophages have been identified as viral targets, leading to inflammatory and metabolic dysregulation [51]. In our model, SHG imaging showed increased collagen deposition in B.1‐infected adipose tissue, indicating variant‐specific tissue remodeling, although H&E staining revealed limited morphological changes. These findings highlight the complexity of host responses to SARS‐CoV‐2 across different organs and underscore the need for longitudinal studies to evaluate fibrotic progression during and after infection. However, metabolic investigation in the adipose tissue with high doses of virus, as used in this study, is compromised by the low amount of fat depots available as diseased mice loses weight. Therefore, further studies should be performed with less viral burden to evaluate the metabolic changes in the adipose tissue. In both tissues, TPEF combined with SHG microscopy uncovered structural alterations not visible with standard H&E staining, highlighting their power in detecting early pathological changes. These advanced imaging techniques may support timely therapeutic interventions, potentially preventing long‐term consequences in COVID‐19 and other diseases.

K18‐hACE2 mice infected with SARS‐CoV‐2 develop a cachexia‐like phenotype, characterized by pronounced body weight loss, depletion of fat depots, metabolic dysfunction, and increased proinflammatory signaling. Thus, we cannot exclude the possibility that the metabolic alterations observed in our study, specifically, the shift toward glycolysis after B.1 infection (as suggested by reduced glycogen stores) and toward lipid oxidation after P.1 infection, are, at least in part, secondary to systemic illness rather than direct viral reprogramming of metabolic tissues. Anorexia is a recognized feature of COVID‐19 [52], and experimental infection of Syrian hamsters with SARS‐CoV‐2 similarly induces weight loss accompanied by increased interferon and TNFα levels [53], a profile consistent with our observations in K18‐hACE2 mice. In humans, COVID‐19 is likewise associated with cachexia and sarcopenia, driven not only by reduced food intake, partly due to anosmia and ageusia, but also by elevated inflammatory cytokines, which are well‐established mediators of anorexia and metabolic dysregulation in chronic disease [54].

Taken together, our findings suggest that systemic factors such as inflammation‐driven anorexia and cachexia may contribute to the metabolic phenotypes observed here. However, the variant‐specific differences between B.1 and P.1, particularly in hepatic metabolic pathways, inflammation, and tissue remodeling, indicate that direct, tissue‐intrinsic effects of viral infection also play an important role. In this context, both B.1 and P.1 SARS‐CoV‐2 variants were capable of infecting key metabolic organs, including the liver and adipose tissue, and inducing distinct pathological and metabolic alterations. Notably, despite similar viral loads, the P.1 variant was associated with more severe clinical outcomes, greater tissue disorganization, enhanced inflammatory gene expression, and a pronounced metabolic shift toward lipid oxidation. These observations highlight the systemic nature of SARS‐CoV‐2 infection and underscore the importance of variant‐specific host responses in driving disease severity and organ dysfunction.

Overall, our study provides new insights into COVID‐19–associated metabolic dysregulation and emphasizes the relevance of extrapulmonary organ involvement in SARS‐CoV‐2 pathogenesis, particularly in the context of emerging variants. Future studies using longer infection timelines or lower viral doses may further clarify the mechanisms underlying SARS‐CoV‐2–induced metabolic alterations, especially in adipose tissue. In this context, the present findings should be interpreted in light of several factors that may influence the results and could be further addressed in future work. The relatively small sample sizes, particularly in the P.1‐infected group due to disease severity, may contribute to variability and limit statistical power. The use of the K18‐hACE2 model, which reflects an acute and severe infection, may not fully capture the complexity and temporal progression of human COVID‐19, including the influence of comorbidities and long‐term outcomes. Furthermore, the pronounced weight loss and cachexia‐like phenotype observed in infected mice may systemically influence metabolic readouts, making it difficult to fully distinguish direct viral effects from secondary consequences of illness. Finally, the use of high viral doses and short infection timelines may impact tissue‐specific analyses, particularly in adipose tissue, and could be refined in future studies to better resolve these effects.

## Author Contributions

**Monara Kaélle Sérvulo Cruz Angelim:** conceptualization, investigation, methodology, validation, formal analysis, data curation. **Patrícia Brito Rodrigues:** methodology, investigation. **Antônio Thiago Pereira Campos:** methodology, investigation. **Giovanni Freitas Gome:** investigation, methodology. **Gabriela Fabiano Souza:** investigation, methodology. **Valquíria Aparecida Matheus:** investigation, methodology. **Carlos Lenz Cesar:** methodology. **José Luiz Proença‐Módena:** methodology, writing – review and editing. **Marco Aurélio R. Vinolo:** methodology, writing – review and editing. **Pedro M. Moraes‐Vieira:** supervision, resources, project administration, funding acquisition, writing – original draft, writing – review and editing, conceptualization. All authors have read and agreed to the published version of the manuscript.

## Ethics Statement

All animal procedures were approved by the Ethics Committee on Animal Use of the University of Campinas (Protocol #5495‐1/2020).

## Conflicts of Interest

The authors declare no conflicts of interest.

## Data Availability

The data that support the findings of this study are available from the corresponding author upon reasonable request.
